# Antimicrobial peptides and proteins of the horse - insights into a well-armed organism

**DOI:** 10.1186/1297-9716-42-98

**Published:** 2011-09-02

**Authors:** Oliver Bruhn, Joachim Grötzinger, Ingolf Cascorbi, Sascha Jung

**Affiliations:** 1 Institute for Experimental and Clinical Pharmacology, Hospitalstraße 4, University Hospital Schleswig-Holstein, Campus Kiel, 24105 Kiel, Germany; 2 Institute of Biochemistry, Olshausenstraße 40, Christian-Albrechts-University, 24098 Kiel, Germany

## Abstract

Antimicrobial peptides play a pivotal role as key effectors of the innate immune system in plants and animals and act as endogenous antibiotics. The molecules exhibit an antimicrobial activity against bacteria, viruses, and eukaryotic pathogens with different specificities and potencies depending on the structure and amino-acid composition of the peptides. Several antimicrobial peptides were comprehensively investigated in the last three decades and some molecules with remarkable antimicrobial properties have reached the third phase of clinical studies. Next to the peptides themselves, numerous organisms were examined and analyzed regarding their repertoire of antimicrobial peptides revealing a huge number of candidates with potencies and properties for future medical applications. One of these organisms is the horse, which possesses numerous peptides that are interesting candidates for therapeutical applications in veterinary medicine. Here we summarize investigations and knowledge on equine antimicrobial peptides, point to interesting candidates, and discuss prospects for therapeutical applications.

## Table of contents

1. Introduction: Antimicrobial peptides - innate antibiotics

1.1. Biological properties

1.2. Peptide characteristics

1.3. Antimicrobial peptide genes

2. Antimicrobial peptides of the horse: history and overview

3. The equine peptides in detail

3.1. Lysozymes

3.1.1. Molecular properties of equine lysozyme and its localization in the horse

3.1.2. Antimicrobial and cytotoxic activity of equine lysozyme and its association with horse disease patterns

3.2. NK-lysins

3.2.1. Equine NK-lysin

3.2.2. Inducibility of NK-lysin by stimulants

3.3. Equine neutrophil antimicrobial peptides (eNAPs) and equinins

3.3.1. eNAP-1

3.3.2. eNAP-2

3.3.3. Equinins

3.4. Psoriasin (S100A7)

3.4.1. The equine psoriasin 1

3.5. Cathelicidins

3.5.1. Equine cathelicidins

3.6. Defensins

3.6.1. Equine β-defensin

3.6.2. Equine α-defensins

3.6.3. Repertoire of equine α-defensins

3.7. Hepcidins

3.7.1. Equine hepcidin

4. Antimicrobial peptides of vertebrates in practice and clinical studies

4.1. Advantages of antimicrobial peptides as therapeutic drugs in general

4.2. Disadvantages of antimicrobial peptides as therapeutic drugs in general

4.3. Antimicrobial peptides of vertebrates in human clinical trials

5. Equine candidates for development of therapeutic applications: capabilities and prospects

5.1. Equine lysozyme

5.2. Equine NK-lysin

5.3. Equine cathelicidins

5.4. Equine β-defensin

5.5. Equine α-defensins

Conclusions

Competing interests

Authors' contributions

Acknowledgments

## 1. Introduction: Antimicrobial peptides - innate antibiotics

Peptides with antimicrobial activities have been known since 1922, when the first lysozyme was observed in human tears by Alexander Fleming and Frederick Ridley [1,2]. Currently over 1700 antimicrobial peptides are known [3] and observed in all kingdoms of life [4-6]. Antimicrobial peptides are an essential part of the innate immune system and act against bacteria, viruses, fungi, parasites, and tumor cells [7,8].

### 1.1. Biological properties

The target specificity, killing efficacy, mode of action, and biochemical properties vary between the peptides. In addition to their antimicrobial activity they can also act as mediators of the adaptive immune system [9] and other cellular processes like wound healing [10]. Most of the peptides exhibit a cationic charge combined with an amphipathic character. They act through an initial electrostatic interaction with the negatively charged compounds of the bacterial cytoplasmic membrane followed by insertion and permeabilization of the membrane. Mostly, membrane integrity is dramatically disturbed resulting in lysis of the targeted microbes [11,12]. However, antimicrobial peptides can also influence intracellular processes through interactions with receptors or signaling molecules and mediate chemotactic or proinflammatory effects. Also by receptor binding some antiviral peptides inhibit the interaction of the virus with the target cell [11,13,14].

### 1.2. Peptide characteristics

Antimicrobial peptides are defined as peptide molecules with an antimicrobial activity, composed of less than 100 amino acids encoded by individual genes. In general they consist of 12 to 50 amino acids including a large proportion of cationic and hydrophobic residues [15]. They can be classified by structural or sequential similarities or by conserved regions on both the amino acid and nucleotide level [11,16,17]. Table 1 exemplifies a classification scheme based on the tertiary structure. Nevertheless, other classification criteria must be used for peptides, whose mature forms possess structural motifs of different classes. In such cases, an amino-acid alignment of precursor peptides is more useful as shown in section 3.5 (cathelicidins).

**Table 1:** Typical structure motifs of mature antimicrobial peptides

Structure motif	α-Helix	β-Sheet	Linear	Loop structure	Cyclic
**Peptide example**	Magainin 2 [188] PDB: 2MAG	Defensin RK-1 [189] PDB: 1EWS	Indolicidin [190] PDB: 1G89	Thanatin [191] PDB: 8TVF	Defensin RTD-1 [192] PDB: 1HVZ

**Ribbon model**					

**Origin**	Frog *Xenopus laevis*	Rabbit *Oryctolagus cuniculus*	Cattle *Bos taurus*	Bug *Podisus maculiventris*	Monkey *Rhesus macaques*

**Disulfide bonds**	-	3	-	1	3

Peptide structures obtained from the Protein Data Bank (PDB).

Antimicrobial peptides are synthesized constitutively or after stimulation by proinflammatory or pathogen associated molecules in circulating phagocytic cells, granulocytes, epithelial cells of mucosal tissues, and glandular cells [8,18]. In many cases they possess an N-terminal signal peptide mediating correct subcellular sorting and trafficking and an anionic propeptide which is thought to be the major contributing factor inhibiting the antimicrobial activity by neutralizing the net positive charge of the mature peptide [19,20]. The propeptides are cleaved by endopeptidases after secretion into the extracellular space (extracellular activation) or before they are incorporated into storage vesicles (intracellular activation) [21,22].

### 1.3. Antimicrobial peptide genes

The anatomy of antimicrobial peptide genes differs widely between peptide families. Most of them consist of 2-5 exons. Genes of structurally related antimicrobial peptides are often arranged in clusters, indicating a common evolutionary ancestor [23-25]. The induction of the antimicrobial peptide gene expression is mostly initiated by binding of transcription factors like NF- κB, AP-1, and STAT3 [26] due to activation of Toll-like receptors by microbial antigens or initiated by cytokines [27].

Mammals are equipped with various antimicrobial peptides. The two major antimicrobial peptide families in mammals are defensins and cathelicidins.

## 2. Antimicrobial peptides of the horse: history and overview

Equine antimicrobial peptides analyzed so far are lysozymes, cathelicidins, defensins, NK-lysin, psoriasin, hepcidin, neutrophilic antimicrobial peptides with homologies to granulins, and equinins. This chapter gives a historical overview of the scientific highlights of the last decades and a basic orientation about the magnitude of the peptides discovered and analyzed.

One of the first equine antimicrobial proteins of the horse, phagocytin, was already described in 1956 by Hirsch [28]. The protein is rich in arginine and therefore possesses a net positive charge. Its antimicrobial activity is pH-dependent and completely inhibited in the presence of albumin. To date we know that these characteristics are typical for antimicrobial peptides, but it remains unclear to which family phagocytin belongs and whether it was rediscovered later and denoted otherwise.

In 1972 Jáuregui-Adell et al. isolated the first equine lysozyme with antimicrobial activity against streptococci from mare milk [29,30]. In the following 18 years no further equine antimicrobial peptides were discovered and only a few investigations with the known milk-lysozyme were performed. But in the 1990s, studies increased substantially starting with the discovery of a second equine lysozyme that was isolated from neutrophil granulocytes and showed a potent antimicrobial activity against Gram-negative and Gram-positive bacteria [31]. In 1992, two additional neutrophil-derived antimicrobial peptides were found, equine neutrophil antimicrobial peptide 1 (eNAP-1) and eNAP-2 [32,33]. Both peptides possess antimicrobial activities against Gram-negative and Gram-positive bacteria, eNAP-2 acts additionally as an inhibitor of microbial proteases [34].

In 1999, Scocchi et al. found three equine cathelicidins in myeloid cells from bone marrow, termed eCATH-1 (equine cathelicidin-1), eCATH-2, and eCATH-3 [35]. Whereas all of these transcripts were found in myeloid cells, only eCATH-2 and eCATH-3 were detected at the peptide level. The synthetic variants were found to be active against various bacteria and fungi.

The first defensin of the horse was identified by Davis et al. in 2004 and named equine β-defensin-1 (eBD-1) [36]. Its transcript is expressed in various tissues. One year later, Davis et al. also identified an equine NK(natural killer cell)-lysin which is specifically expressed in lymphocytes [37]. The biological activities of eBD-1 and NK-lysin are unknown. Also in 2005, the mRNA-sequence of the equine psoriasin (a calcium-binding protein of the S100-protein family with antimicrobial activities [38]) was described and the gene was mapped to equine chromosome 5 [39].

A putative α-defensin and additional β-defensins in the genome of the horse were detected in 2006 by analyzing the sequence of an equine BAC-clone [40]. A comprehensive analysis of the defensins was performed in the following years [41,42]. The antimicrobial activities and the mode of action of the first equine α-defensin DEFA1 were investigated. In 2009, Bruhn et al. analyzed the full repertoire of equine α-defensin transcripts in the intestine and found 38 different equine α-defensin transcripts [41]. The high number of potentially active α-defensins is remarkable in mammalia, and within the subgroup of *Laurasiatheria *the *Equidae *are the only known family expressing α-defensin genes.

In 2010, two cathelicidin-derived antimicrobial peptides were found in the donkey (*Equus asinus*) denoted as EA-CATH1 and EA-CATH2 [43]. The chemically synthesized peptide variants exhibit antimicrobial activities against bacteria and fungi. Finally, equine hepcidin, an antimicrobial peptide also involved in iron homeostasis, was discovered and analyzed concerning its tissue distribution.

## 3. The equine peptides in detail

**Figure 1:**
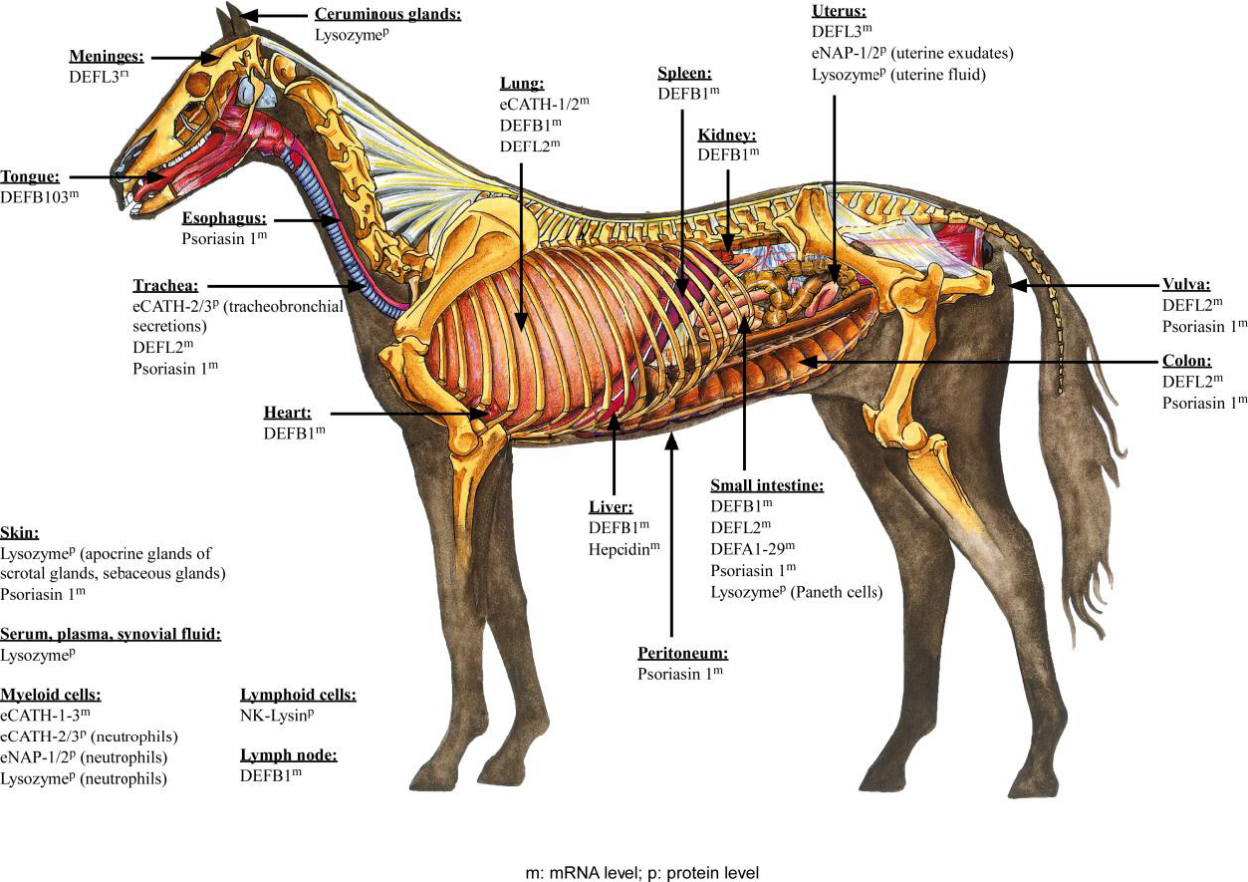
**Expression of equine antimicrobial peptides in different tissues of the horse**. Expression was detected on the mRNA-level or the protein level, respectively, as indicated by superscript characters *m *and *p*. The picture "Internal organs of the horse" was provided with kind permission by Virbac (^© ^Virbac).

Equine antimicrobial peptides have been detected on mRNA or protein level, respectively, in numerous tissues of the horse (Figure 1). Characteristics of the peptides are summarized in table 2. The following chapters are introduced by a description of the corresponding peptide family in general.

**Table 2:** Antimicrobial activities and characteristics of equine antimicrobial peptides

Peptide family Peptide	Mass [kDa]	Charge (at pH 7.5)	Specificity	Salt dependency	Citation
**Cathelicidins**					
					
eCATH-1 [GenBank: CAA12226]	3.1	8.8	Gram-positive/negative bacteria Fungi	no no	[35,111]
eCATH-2 [GenBank: CAA12227]	3.6	2.8	Gram-positive/negative bacteria Fungi	no yes	[35,111]
eCATH-3 [GenBank: CAA12228]	4.7	7.0	Gram-positive/negative bacteria Fungi	yes yes	[35,111]
eCATH-4 [GenBank: XP_001495199.2]	n. d.	n. d.	n. d.		this review
EA-CATH-1 [GenBank: ACN93988.1]	3.1	6.8	Gram-positive/negative bacteria Fungi	no no	[43]
					
**Defensins**					
					
DEFB1 [GenBank: AAO32801]	4.0	5.2	n. d.		[36]
DEFL2 [GenBank: CAJ01792]	4.5	6.5	n. d.		[40,145]
DEFL3 [GenBank: CAJ01793]	4.2	5.6	n. d.		[40,145]
DEFB103 [GenBank: CAJ01801]	4.7	9.2	n.d.		[40,145]
DEFA1 [GenBank: ABP96800]	4.1	6.3	Gram-positive/negative bacteria Yeast	yes yes	[40-42,131]
DEFA2 to 29 [GenBank: ACV49728-ACV49755]	3.6 - 5.5	2.3 - 9.2	n. d.		[41]
					
**Neutrophil antimicrobial peptides**					
					
eNAP-1 [GenBank: AAB22706]	7.2	n. d.	Gram-positive/negative bacteria	n. d.	[33]
eNAP-2 [GenBank: AAB24353]	6.5	n. d.	Gram-positive/negative bacteria	n. d.	[32,34]
					
**Lysozymes**					
					
Equine lysozyme [Swiss-Prot: P11376]	14.7	2.2	Gram-positive/negative bacteria	n. d.	[46-48,50]
					
**NK-lysins**					
					
Equine NK-lysin [GenBank: AAN10122]	9.2	7.2	n. d.		[37,82]
					
**Psoriasin (S100A7)**					
					
Psoriasin 1 [GenBank: CAH03717]	11.3	-6.2	*Escherichia coli*	n.d.	[39,100]
					
**Hepcidin**					
					
Equine hepcidin [GenBank: XM_001491610.2]	2.8	1.1	n. d.		[158]

Peptide masses and charges that have not been determined experimentally were complemented using the ProteinCalculator v3.3 software [193].

### 3.1. Lysozymes

Lysozymes are glycoside hydrolases that cleave β-(1,4)-glycosidic bonds of bacterial cell walls [44]. Three types of lysozymes can be distinguished: c-type (chicken or conventional type), g-type (goose-type) and i-type (invertebrate type) lysozyme. These types differ in their primary structure and in their biochemical properties but their tertiary structures own the same topology. The first lysozyme tertiary structure was determined from hen egg white by X-ray crystallography [45]. It shows two domains of α-helical or β-strand character, respectively, that harbor the active site between them. The in vivo antibacterial activity of the lysozymes is limited by numerous general cell-wall modifications hindering the enzyme to reach its substrate [44]. This is compensated in vivo by a simultaneous attack of a cocktail of components (defensins, cathelicidins) that disrupt the membrane integrity. The more specialized lysozyme inhibitors seem to be restricted to c-type lysozymes. Nevertheless, by cleaving the major bacterial cell wall component peptidoglycan lysozymes contribute to the antibacterial defense, considerably.

#### 3.1.1. Molecular properties of equine lysozyme (EL) and its localization in the horse

The equine lysozyme consists of 129 amino acids and possesses essential features of chicken-type lysozymes [46]. It shows 50-51% sequence identity with human milk lysozyme and with domestic hen egg white lysozyme. Interestingly, it was found that EL binds calcium ions in an equimolar ratio, whereas human and hen egg white lysozyme do not [47,48]. As lysozymes are homologous proteins of α-lactalbumins which can bind calcium, EL is seen as an evolutionary link between lysozymes and α-lactalbumins [49,50]. The binding of calcium by EL influences its molecular structure and participates in EL oligomer formation [51-53]. Structural studies of EL, its calcium binding property and the EL oligomer formation are reviewed in [50]. Two things have to be mentioned: first, the tertiary structure of EL was never determined de novo, but deduced from modeling or molecular replacement, respectively [48,54,55]. Second, very recently an analysis of its calcium/magnesium selectivity revealed a distinct additional magnesium-specific site in EL [56].

Many of the EL data summarized in this review originated from mare-milk lysozyme. However, there are several other loci where lysozyme can be detected in the horse, e. g. serum and plasma [57-60], synovial and uterine fluids [59,61], intestinal Paneth cells [62,63] and granulocytes [31] (Figure 1). Besides the secretory granules of Paneth cells, EL was localized on the subcellular level in the secretory granules, Golgi apparatus, and elements of the rough endoplasmic reticulum of apocrine glands of the equine scrotal skin [64].

#### 3.1.2. Antimicrobial and cytotoxic activity of equine lysozyme and its association with horse disease patterns

The antimicrobial activity spectrum of EL includes Gram-positive bacteria as *Streptococcus (Strep.) equines *[30], *Micrococcus luteus*, *Bacillus subtilis*, and *Staphylococcus (Staph.) lentus *[31]. *Staph. aureus *and *Staph. epidermidis *were not lyzed. Moreover it was found to be bactericidal against the Gram-negative bacteria *Escherichia (E.) coli*, *Klebsiella (Kl.) pneumoniae*, *Bordetella bronchiseptica*, and *Serratia (Ser.) marcescens*.

The enzymatic activity of EL is dependent on several factors. Whereas EL is still working after heating to 100°C at acidic and neutral pH, its activity is markedly affected by alkaline pH [65]. Interestingly, in female Arabian horses, the activity of the enzyme was shown to follow a seasonal periodicity [66]. Lysozyme activity in milk during lactation revealed highest activities in the first three days post partum [67]. This activity rapidly decreased until the 9^th ^day. In this regard, race and time of conception revealed to be significantly important.

EL is able to form oligomers that can assemble to linear or annular protofilaments [68]. Whereas the monomers and the protofilaments are harmless, the oligomers are cytotoxic and induce cell death in primary neuronal cells, fibroblasts, and neuroblastoma cells [69]. Recently, EL was found to assemble into multimeric complexes with oleic acid (ELOA) which are also cytotoxic [70].

The association of equine lysozyme with several disease patterns in the horse is proven by different studies mainly by descriptive investigation of its expression level. In surgically induced cartilaginous defects in the radiocarpal joints of horses, the synovial fluid lysozyme concentration was found to be significantly increased and of lysosomal origin [59]. Furthermore, the increased lysozyme concentration was correlated positively with increased numbers of leukocytes in the synovial fluid. Whereas the joint defects did not influence the plasma lysozyme, in myelomonocytic myeloproliferative disease the concentration of EL was almost three times increased [57]. The plasma lysozyme activity was also investigated in experimental fever induced by administration of *E. coli *lipopolysaccharide (LPS) to tarpan-like horses. Three hours after injection of LPS, the plasma lysozyme level was significantly increased [60]. The association of lysozyme with inflammation was also observed with experimentally induced bacterial endometritis. Lysozyme levels progressively increased in uterine flushings with time after infection [61]. During acute equine alimentary laminitis EL was secreted by degranulation of Paneth cells in response to the deregulation of the microbial balance in the gut. This finding suggests the contribution of this enzyme to the mucosal defense system of the equine intestinal tract [62]. The serum EL-concentration in older horses (average age: 19 yr) after vaccination with virus was significantly changed depending on vitamin-E supplementation. Supplemented animals did not show an increase in contrast to placebo-treated horses exhibiting a continuous increase of serum EL [71]. Finally, EL is proposed to support the function of the cerumen as a non-specific antimicrobial agent in the external auditory canal [72].

### 3.2. NK-lysin

NK-lysin was first isolated from porcine small intestine showing high antibacterial activity against *E. coli *and *Bac. megaterium *[73]. It is a primary effector molecule of cytotoxic T- and NK-cells [73,74]. NK-lysins are inducible by cytokines, have a lytic activity against eukaryotic cells except red blood cells, and additionally possess a tumorolytic activity [73,75]. They were identified in cattle [76], chicken [77], flounder [78], channel catfish [25], horses [37], and *Fasciola hepatica *[79]. A related protein family in humans is denoted as granulysins [80]. Interestingly, NK-lysins show a homology to the amoebapores derived from *Amoeba *indicating an evolutionary connection between the leukocytes of higher animals and their unicellular protozoan ancestors [81].

#### 3.2.1. Equine NK-lysin

**Figure 2:**
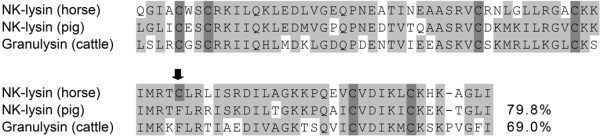
**Comparison of mature NK-lysins and granulysin from farm animals**. Primary structure alignment of mature NK-lysins from the horse and pig and granulysin from cattle. Conserved and semi-conserved amino-acid residues with similar properties are highlighted in light grey, cysteine residues in dark grey. Similar amino acids were defined as follows: (A, G); (S, T); (E, D); (R, K, H); (Q, N); (V, I, L, M); (Y, F); (W); (P); (C) [81]. The arrow indicates an additional cysteine residue only present in the equine sequence. Gaps introduced to maximize amino-acid alignments are indicated by hyphens. The percent similarity to equine NK-lysin is given at the end of the pig/cattle sequence.

The equine NK-lysin was described by Davis et al. in 2005 [37]. Its amino-acid sequence showed a similarity of 80% compared with the porcine homolog and 69% with the bovine homolog (Figure 2). The equine mature peptide consists of 83 amino acids. Six cysteine residues that are in general associated with the antimicrobial activity of the peptide are conserved between the equine, porcine and bovine primary structure. An additional seventh cysteine residue can be found only in the equine amino-acid sequence.

The transcription profile of the equine NK-lysin mRNA was analyzed in more detail by separating the peripheral blood mononuclear cell population. A transcript of the equine NK-lysin was identified in lymphoid cells but not in granulocytes. The authors concluded that equine NK-lysin is ubiquitously expressed in equine lymphocytes as for NK-lysins of other species [37].

To determine whether equine NK-lysin gene expression is inducible, mononuclear cells were examined after stimulation with concanavalin A (a lectin that stimulates lymphocytes). Treatment of lymphocytes with 1 μg of the mitogen leads to an increase in gene expression of approximately 50% [37]. The antimicrobial activities of the equine NK-lysin have not been determined up to now.

#### 3.2.2. Inducibility of NK-lysin by stimulants

Davis et al. could show that treatment of horses with *Propionibacterium (Pb.) acnes *leads to an enhanced gene expression of IFN-γ and NK-lysin in peripheral blood mononuclear cells [82]. Commercially available non-viable *Pb. acnes *cells can be used as immune stimulants that activate macrophages and mononuclear cells, contributing to an enhanced elimination of pathogens [83]. Immunostimulation in horses with *Pb. acnes *led to an increased number of CD4^+ ^lymphocytes and an enhanced phagocytosing activity and lymphokine-activated killing [84].

These studies underline the beneficial responses of *Pb. acnes *immunostimulation in horses and show that antimicrobial peptides are not only interesting as drugs, but also as endogenous agents that can be stimulated by immunomodulatory substances to promote the immune system.

### 3.3. Equine neutrophil antimicrobial peptides (eNAPs) and equinins

Equine neutrophil derived antimicrobial peptides include cathelicidins, lysozymes, eNAP-1, and eNAP-2. Interestingly, neutrophils of the horse lack defensins that depict mostly the main peptide type of these cells in other species. The equine neutrophil peptides eNAP-1 and eNAP-2 do not share sequential similarities and are included in different peptide families [33]. Interestingly, both of these families are not known as typical antimicrobials. Equine NAP-1 shows a similarity to the peptide-group of granulins, mainly known as cytokines of different species [33]. In contrast, eNAP-2 shows a homology to a peptide group of equine microbial protease inhibitors denoted equinins. In the original publication of Couto et al. eNAP-2 was related to the rat WDNM1 protein which is included in a four-disulfide core family of proteins with diverse functions and a homology to human antileukoproteases [32].

#### 3.3.1. eNAP-1

Approximately 0.5 μg of eNAP-1 and up to 5 μg of eNAP-2 were isolated from 10^9 ^polymorphonuclear leukocytes (PMN), which represents comparatively only 1% of the amount of stored defensins in human neutrophils [33]. Defensins were absent in equine PMN.

Equine NAP-1 contains 10 cysteine residues that are completely conserved compared to the peptide family of granulins isolated from human neutrophils [85]. Whereas granulins are known as growth factors and cell communication molecules with diverse biological functions but without antimicrobial activities, eNAP-1 showed an antimicrobial activity against three of four tested clinical isolates of common equine uterine pathogens: *Strep. equi *subsp. *zooepidemicus*, *E. coli*, and *Ps. aeruginosa*. A bacteriostatic activity was observed against *Kl. pneumoniae *[33]. However, compared with the antimicrobial activities of, e.g., defensins, eNAP1 possesses a rather weak antimicrobial activity.

#### 3.3.2. eNAP-2

As in the case of eNAP-1, the amino-acid analysis of eNAP-2 also revealed high cysteine content [32]. Its antibacterial activity was assessed with the same equine pathogens that were already used for eNAP-1. The reduction of the input CFU/mL was 94% for *Strep. equi *subsp. *zooepidemicus *at a peptide concentration of 100 μg/mL, 90.2% in the case of *E. coli*, and 77.6% for *Ps. aeruginosa*. *Kl. pneumoniae *was unaffected. These antimicrobial activities were comparable to eNAP-1, but to some extent lower against the tested Gram-positive strain *Strep. equi *subsp. *zooepidemicus *[32]. However, whereas eNAP-1 is present only in very low quantities in equine neutrophils, eNAP-2 seems to be one of the major gene products [32].

Equine NAP-2 was shown to be related to a peptide called WDNM1 [86]. Five cysteine residues were highly conserved between these peptides. WDNM1 is present in rats, mice (also known as Expi, Kal1), and cattle. It acts as an extracellular proteinase inhibitor and is associated with cell differentiation processes, apoptosis and cancer [87,88]. Couto et al. showed the relationships between eNAP-2 and several four-disulfide core antiproteases and also the relationships between eNAP-2 and equine cationic peptides exhibiting selective inhibitory activity against subtilisin and proteinase K (later denoted equinins), reported by Pellegrini et al. in 1988 [89]. In a follow-up study, Couto et al. demonstrated the selective inhibition of microbial serine proteases by eNAP-2 [34]. Equine NAP-2 inhibited the microbial proteinases subtilisin A and proteinase K and did not affect the enzymatic activity of human neutrophil elastase, human cathepsin G, or bovine pancreatic trypsin. Additionally, it was demonstrated that eNAP-2 formed an enzyme-inhibitor complex with subtilisin A and proteinase K but not with other tested proteinases. The authors concluded that the dual antimicrobial and antiproteinase activities of eNAP-2 might attribute the peptide an important role in equine host defense against infections [34].

#### 3.3.3. Equinins

The antimicrobial activity of equinins (with the exception of eNAP-2) has not yet been proven, but they are able to selectively inhibit the microbial proteinases subtilisin and proteinase K. Equinins were discovered by Pellegrini et al. in 1988 [89,90] and denoted as low-molecular weight proteinase inhibitors/isoinhibitors from the granular fraction of equine neutrophilic granulocytes.

Five potential proteinase inhibitors isolated from the granule-rich sediment of disrupted neutrophils were purified using chromatography techniques. The molecules separated with molecular weights of 6.3 to 11.3 kDa were tested for their inhibitory activities against 13 different proteinases, but all five equine neutrophilic proteinase inhibitors inhibited exclusively proteinase K and subtilisin. Thus, the inhibitory activities are only directed against microbial proteases and not against granule proteinases, suggesting that they may act as endogenous antibiotics [90]. In fact, the direct antimicrobial activities of the proteinase inhibitors were not determined to date, but it is possible that the inhibitors could interfere with acyl-serine proteases involved in bacterial cell wall synthesis.

The equine neutrophilic proteinase inhibitors share their target specificity with eNAP-2, but in contrast they were stable at temperatures up to 100°C and the inhibitory activity remained constant at a broad pH-range. The membership of eNAP-2 to the proteinase inhibitors, reported by Couto et al. [34], was confirmed by Pellegrini et al. some years later [91]. They combined eNAP-2 and the equine neutrophilic proteinase inhibitors to one group denoted equinins.

### 3.4. Psoriasin (S100A7)

Psoriasin (S100A7) is a member of the S100-gene family that represents the largest subgroup of the calcium-binding protein super-family [92]. The characteristic structural motifs of the S100-gene family are two EF-hands, comprised of a helix-loop-helix unit, that are able to bind bivalent ions like calcium and zinc. Psoriasin was first identified as a secreted protein in extracts of human skin from psoriasis patients [93]. It is synthesized in keratinocytes and is highly upregulated in psoriatic skin [94] and epithelial skin tumors [95]. It was shown that psoriasin is a potent and selective chemotactic inflammatory protein for CD4^+ ^T-cells and neutrophils [96]. The protein was identified as the major *E. coli *killing factor in both healthy and psoriatic skin, the human tongue, and the female genital tract [38,97,98]. Psoriasin acts by membrane permeabilization, depending on the pH-value [99]. Members of the S100 family have been identified in all vertebrates observed to date and are clustered in the genome.

#### 3.4.1. The equine psoriasin 1

The equine *S100A7 *gene, called psoriasin 1, was confirmed and assigned to chromosome 5p12-p13 in 2005 [39]. Its genomic sequence indicates that one intron of approximately 1.1 kbp separates two exons. Comparative studies with sequences from different species showed the highest identities with human *S100A7 *(85%) and *S100A7 *of *Bos taurus *(80%). A transcriptional analysis showed the existence of equine psoriasin 1 transcripts in skin, trachea, esophagus, intestine, and vulva [100].

The equine psoriasin was synthesized recombinantly and different oligomers were isolated and analyzed regarding their antimicrobial activities and mode of action. A weak antimicrobial activity against *E. coli *was observed for the dimer as well as a weak pore-forming activity [100].

### 3.5. Cathelicidins

The first member of the cathelicidin family was isolated from cattle in 1993 [101]. The term cathelicidins indicates the evolutionary relationship of this protein family to the cathelins. Cathelicidins consist of an N-terminal cathelin-like sequence (~ 120-150 amino acids) and a C-terminal cationic antimicrobial domain (~10-95 AA) [102,103]. The antimicrobial domain, which is the mature cathelicidin peptide, becomes active after cleavage from the N-terminus. Cathelicidins are stored as holoproteins, precursors that lack antimicrobial activity. They are synthesized predominately in myeloid cells and are stored in secretory granules of neutrophils. They were also found in different epithelia, organs, and secretions of glands (reviewed by [103] and [104]).

**Figure 3:**
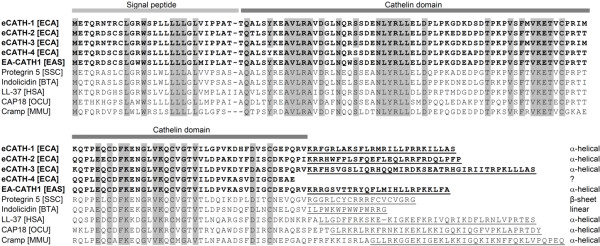
**Amino-acid sequence alignment of selected mammalian cathelicidin precursors**. Equine cathelicidins are shown in bold letters. Conserved amino-acid residues are highlighted in grey. Mature peptides are underlined. The peptide structure of the mature peptides is given at the end of each sequence. The signal peptide (light grey) and the cathelin domain (dark grey) are indicated by lines on top of the sequence block. Species abbreviations: [ECA] = *Equus caballus*, [SSC] = *Sus scrofa*, [BTA] = *Bos taurus*, [HSA] = *Homo sapiens*, [OCU] = *Oryctolagus cuniculus*, [MMU] = *Mus musculus*.

In contrast to most of the other families of antimicrobial peptides, mature cathelicidins are highly heterogeneous. The reason for combining these diverse molecules to one family is the highly conserved N-terminal cathelin domain of the precursor protein (Figure 3). Most of the mature cathelicidins are linear peptides that adopt an α-helical fold with an amphipathic character when exposed to hydrophobic environments (e.g., biological membranes), but β-sheet structures stabilized by disulfide bonds, loop structures or extended polyproline structures are also known [104].

Cathelicidins were observed only in vertebrates to date. Regarding mammals, their existence was demonstrated in humans, monkeys, rabbits, rats, mice, guinea pigs, pigs, cattle, sheep, goats, and horses [104]. Intensively investigated cathelicidins are protegrins from swine, indolicidin from cattle, human LL-37, CAP 18 from rabbits, and CRAMP from mice [103,105].

Cathelicidin genes span about 2 kb and contain four exons. The fourth exon codes for the mature peptide [103]. Each species contains a different set of cathelicidin genes that are clustered in the genome, e.g., the bovine genome contains eleven cathelicidin genes [24], eight genes were found in sheep [106], and only one cathelicidin gene in humans.

Cathelicidins are microbicidal against Gram-negative and Gram-positive bacteria [107], fungi [108], enveloped viruses [109], parasites [108], and tumor cells [110]. Unlike most defensins, many cathelicidins display a bactericidal, antiparasitic, and antiviral activity at physiological salt concentrations and are also active in the presence of serum [103,104].

Permeabilization of bacterial membranes is a common mode of action of cathelicidins. The antiviral activities emerge through interactions of the peptides with viral receptors or by disrupting the integrity of the viral envelope. Additionally, they have chemotactic effects and are able to recruit circulating leukocytes, neutrophils, and monocytes. Cytotoxic effects have been reported for some cathelicidins against eukaryotic cells (hematopoietic cell lines, erythrocytes, and proliferating cells) [103,104].

#### 3.5.1. Equine cathelicidins

Three cathelicidins of the horse have been investigated to date, eCATH-1, eCATH-2, and eCATH-3 (table 2) [35,111]. The cathelicidins were identified using RNA from bone marrow [35]. The cDNA sequences of the horse share 92-99% identity in the 5'-region and are diverse at the mature peptide coding region (Figure 3). The sequence of eCATH-3 is longer than those of eCATH-1 and eCATH-2. Additionally, eCATH-3 comprises a short interspersed nucleotide element in the 3'-UTR flanked by direct repeats and AT-rich microsatellites. The presence of a retroposon and microsatellite sequences in the eCATH-3 gene suggests that the genomic region is unstable and may account for the generation of genetic variations [35]. Investigations of the mRNA-expression levels of the three cathelicidins in myeloid cells revealed an abundant expression of eCATH-2 and e-CATH-3 and a significantly lower level of eCATH-1.

Genetic analysis with more than 70 horses revealed that 50% of horses carry null alleles of eCATH-1 [35]. Polyclonal antibodies against all three eCATH variants were generated using synthetic eCATH peptides. Western blotting analysis revealed the existence of eCATH-2 and eCATH-3 peptides in all horses analyzed. On the contrary to expectations, the eCATH-1 antibodies failed to recognize eCATH-1 in both neutrophils and neutrophil granule lysates. It was suggested that the eCATH-1 gene is unable to encode a protein [35]. This finding was confirmed by analyzing the ability of equine neutrophils to secrete eCATH peptides. The analysis consistently revealed only the presence of eCATH-2 and eCATH-3, further supporting that the eCATH-1 gene is non-functional.

Insights into the processing of the peptides were accumulated by analyzing different samples of tracheobronchial secretions of horses affected by chronic obstructive pulmonary disease (COPD) and acute bronchiolitis [111]. Only eCATH-2 and eCATH-3 were detected, being consistent with the observations of the first study. Elastase was identified as the enzyme responsible for processing cathelicidins of the horse.

The structure and activities of the three eCATH were examined using synthetic peptides that corresponded to the deduced sequences. Investigation of the secondary structures by circular-dichroism spectroscopy confirmed α-helical contents of approximately 46.5% for all three peptides in the presence of a helicogenic solvent. In an aqueous solution only eCATH-2 shows an α-helical content of 22.7%. When displayed as a helical wheel, all three peptides displayed moderate amphipathicities [111].

The most bactericidal cathelicidin is the synthetic eCATH-1 (that is not present in vivo) with minimal inhibitory concentrations (MICs) between 3 and 12 μg/mL for Gram-negative bacteria (including *E. coli*, *Salmonella enterica*, *Ps. aeruginosa*, *Kl. pneumoniae*, and *Ser. marcescens*) and between 6 and 24 μg/mL for Gram-positive bacteria (including *Staph. aureus*, *Staph. epidermidis*, *Strept. equines*, and *Bac. megaterium*). Equine CATH-2 was active against *E. coli *(14 μg/mL), *Ser. marcescens *(28.5 μg/mL), *Kl. pneumoniae *(28.5 μg/mL), *Staph. aureus *and *Staph. epidermidis *(57.0 and 28.5 μg/mL), and *Bac. megaterium *(14 μg/mL). Surprisingly, eCATH-3 proved to be ineffective against all bacterial strains up to peptide concentrations of 150 μg/mL. An antibacterial activity was only observed when testing eCATH-3 in low-ionic-strength medium [111].

Marked differences in antifungal activity were also noticed. No equine cathelicidin showed activity against *Candida *spp. or *Pichia etchellsii*. *Cryptococcus *spp. and *Rhodotorula rubra *were sensitive to synthetic eCATH-1. Equine CATH-2 only showed antifungal activities at physiological salt concentrations, whereas CATH-3 was only active in low salt medium. Based on previous reports that point to a correlation between amphipathicity and antimicrobial activity [112], a slightly modified eCATH-3 peptide was synthesized (LLK-eCATH-3) with a higher hydrophobic moment and helical content realized by the substitution of three amino-acid residues. These modifications caused a dramatic change in the antimicrobial activity. Antibacterial activities of LLK-eCATH-3 were comparable with that of eCATH-1 (which was the most potent eCATH) and antifungal effects were also noted at physiological salt concentrations [111].

By reflecting the in vitro-data of these peptides some questions inevitably arise. Curiously, the most potent peptide in vitro (eCATH-1) is not present in equine myeloid cells. However, an (increased) eCATH-1 expression might occur only under particular conditions, e.g., after stimulation by microbial antigens. Moreover, it cannot be excluded that eCATH-1 might be expressed in other cell types than myeloid cells as demonstrated for other members of the cathelicidin family, e.g., the lung [113], squamous epithelia [114], or in cancerous tissues [115]. Another point is the modest in vitro-activity of eCATH-3, especially under physiological salt concentrations, even though the peptide is present at high levels in vivo. Different suggestions were made: first, the abundance of the peptide at sites of release should be taken into account. This is a common, but plausible statement to explain low in vitro-activities or salt sensitivities, because most of the antimicrobial peptides are produced for acting in short time intervals and after release from storage granules the peptides can locally reach remarkable high amounts as known from defensins [116]. Additionally, eCATH-3 was found in inflammatory secretions, indicating that the peptide can also be released in extracellular fluids.

In addition to the cathelicidins 1 to 3, a fourth cathelicidin sequence exists in the genome of the horse, recorded and predicted by automated computational analysis (NCBI's Annotation Process: "similar to myeloid cathelicidin 2"). The sequence seems to be incomplete. However, the predicted amino acid sequence (denoted eCATH-4 in this review) is included in Figure 3 and the corresponding gene information in Table 2.

Recently, the existence of novel cathelicidin-derived antimicrobial peptides from *Equus asinus*, the African donkey, has been described (denoted EA-CATH1 and EA-CATH2) [43]. The peptides were identified from a lung cDNA library, suggesting that the eCATH-peptides could possibly be found in tissues next to myeloid cells. The mature peptides are composed of 25 and 26 residues comparable with eCATH-1 and eCATH-2. Chemically synthesized EA-CATH1 exerts a potent antimicrobial activity in the range of 0.3-2.4 μg/ml against various bacteria and fungi, including clinically isolated drug-resistant strains. EA-CATH1 is also active at physiological conditions and does not show cytotoxic activity at concentrations up to 20 μg/mL (actually, this is a very low concentration for testing hemolytic activities). EA-CATH1 possesses an α-helical conformation in an acidic milieu.

The authors performed scanning-electron microscopy to determine the mode of action of EA-CATH1 using *Staph. aureus *as a model organism. The analysis revealed a rapid disruption of the bacterial membrane. In addition, the authors observed a possible restriction of bacterial colonization and spread caused by an exerted hem-agglutination activity of EA-CATH1 in the presence of CaCl_2_, which might potentiate an inhibition against bacterial interactions with the host erythrocyte surface [43]. EA-CATH2 did not show antibacterial activity.

### 3.6. Defensins

The defensins represent the major class of antimicrobial peptides in vertebrates. They are comprehensively reviewed in [117-119]. Defensins are cationic and cysteine-rich peptides with molecular masses ranging from 3 to 5 kDa. The mature peptide consists of 18 to 45 amino acids and contains six highly conserved cysteine residues forming characteristic intramolecular disulfide bonds. The disulfide array is specific for the three defensin sub-families in mammals: α-, β-, and θ-defensins [117]. Alpha- and β-defensins consist of three antiparallel β-sheets, whereas some β-defensins possess additionally an N-terminal α-helix. Next to biochemical differences between the defensin subgroups, there are crucial varieties regarding their biology. An α-defensin synthesis is presumably unique to a few tissues. They were found in neutrophils [120], epithelial cells of the human female urogenital tract [121], the kidney of rabbits [122], and in the intestinal Paneth cells of different species [123]. Furthermore, their synthesis is limited to some species, they were exclusively found in mammals (also basic ones such as platypus) except cattle [124] and dogs [125]. In contrast, β-defensins were observed in vertebrates, arthropods, mollusks, and plants, where they are expressed in various tissues [117]. The somewhat unusual θ-defensins exhibit a cyclic β-sheet structure which is a result of a posttranslational ligation of two truncated α-defensin precursors [126]. Functional peptides were only verified in non-human primates, whereas in all other mammals the truncated α-defensin precursors have additional premature stop codons and are pseudogenes [127,128].

Defensins are synthesized in vivo as inactive precursor proteins. Proteolytic excision of the N-terminal inhibitory anionic propeptide is required for maturation and activation [19,20]. The mature defensins exhibit an antimicrobial activity against a broad spectrum of microorganisms including Gram-negative and Gram-positive bacteria [117], fungi [13,129], and enveloped viruses [130] in a concentration range of 0.1 to 5.0 μM. They kill bacteria through an initial electrostatic interaction with the negatively charged phospholipids of the microbial cytoplasmatic membrane, followed by membrane permeabilization, and finally lysis of the microbes [6,11].

The antimicrobial activity of most of the defensins was reduced at increasing salt concentrations or in the presence of bivalent cations [131]. However, physiological concentrations of 1 to 10 mg/mL peptide were measured in leukocytes and in the lumen of crypts of Lieberkühn and have been shown to kill microorganisms in vitro at physiological salt concentrations [132]. Secretion of defensins can be constitutive or induced by prokaryotic antigens through a Toll-like-receptor mediated process [133]. In addition, an induction after cytokine stimulation was described which is often mediated by the transcription factors NF- κB and STAT3 [26,27,134]. Moreover, defensins are agonists for cytokine or chemokine receptors and represent a link between the innate and adaptive immunity [135-137].

The genetic anatomy of α- and β-defensins exhibits a conserved exon/intron-structure. They mostly consist of two or three exons, respectively. The last exon codes for the mature peptide. Defensin genes are arranged in clusters. The number of individual genes varies widely between species [41,138-140]. It is assumed that the species-specific composition of defensin genes is a result of evolutionary duplications and deletions from single genes or genomic regions of the defensin cluster [141,142]. The origin of the ancestral defensin gene is not finally clarified [143,144].

#### 3.6.1. Equine β-defensins

The first β-defensin of the horse was identified by Davis et al. in 2004 [36]. It was denoted β-defensin-1 (later renamed *DEFB1*). The derived amino-acid sequence (peptide: eBD-1) shows the typical conserved cysteine residues. Comparison with the cDNA sequences of other β-defensins revealed 45-52% similarity to the bovine, caprine, and porcine homologs, respectively. The closest similarities were observed between eBD-1 and human BD-2 or porcine BD-1.

*DEFB1 *expression was detected in different horse tissues (Figure 1). Therefore, a constitutive expression was assumed. The amplified products were not analyzed by sequencing. Thus, it cannot be excluded that other β-defensins or defensin-related products among *DEFB1 *were amplified. However, Davis et al. clearly demonstrated expression of β-defensins in a variety of horse tissues [36].

The determined *DEFB1 *sequence was used to screen the equine CHORI-241 BAC library [40]. The equine BAC clone CH241-245H5 was selected for sequencing. The clone comprised approximately 213 kb and was mapped to ECA27q17. The sequence contained eight potentially functional defensin genes (seven β-defensins and one α-defensin gene), and five defensin-related pseudogenes. The α-defensin was denoted *DEFA5L *(alpha-defensin 5 like) because of the high similarity to the human α-defensin *DEFA5*. Similarities of the β-defensins were found with canine and rodent specific genes (*DEFL1 *to *DEFL3*), as well as human β-defensins *DEFB4*, encoding hBD3 (*DEFB1 *to *DEFB3*), and *DEFB3*, encoding hBD3 (*DEFB103*). The defensin-related pseudogenes show premature stop codons, have start-codon mutations or miss different conserved amino acids.

The authors demonstrated that equine β-defensins are similarly organized as in other mammals and show the typical genomic clustering [40]. A limitation of the study was that all these sequences were not confirmed by transcriptional analysis or by sequencing of the corresponding equine cDNA.

A transcriptional analysis with equine *DEFB1 *was already performed by Davis et al. [36]. *DEFB2 *as well as *DEFB3 *are duplicates of *DEFB1 *[40]. The remaining β-defensins (*DEFL1*, *DEFL2*, *DEFL3*, *DEFB103*) and the α-defensin (*DEFA5L*) were analyzed by Bruhn et al. in 2008 using RNA from 14 different tissues of the horse [145]. The existing transcripts were verified for all defensins except *DEFL1 *(Figure 1). Interestingly, *DEFB103 *was exclusively transcribed in the tongue, indicating a special oral defensin. The human *DEFB103 *is also expressed in oral epithelia and the gene product hBD-3 has prominent antimicrobial activities.

Besides genomic studies and transcription analyses, equine β-defensins were directly detected in glands and glandular secretions. As lysozyme, β-defensins were demonstrated to be products of the equine apocrine ceruminous- and sebaceous-glands and are suggested to be involved in the function of cerumen as a general antimicrobial protective agent in the external auditory canal [72]. Two years later the detailed subcellular localization of lysozyme and β-defensin in the apocrine glands of the equine scrotal skin was analyzed [64]. Antimicrobial β-defensins were localized in the secretory granules, the Golgi apparatus and in the cisternae of the rough endoplasmatic reticulum. It was suggested that the presence and secretion lead to a protective effect and a non-specific defense against microorganisms exhibited by the apocrine glands.

#### 3.6.2. Equine α-defensins

The potentially functional α-defensin (*DEFA5L*) on the equine BAC clone CH241-245H5 [40] was duplicated once producing the pseudogene *DEFA5LP*. The term *DEFA5L *is based on the high identity (80.1%) to the human Paneth cell-specific α-defensin *DEFA5*. Further striking similarities were observed with the rat neutrophil peptide NP-2 (82.8%) and α-defensin 4 of the rhesus monkey (79.1%). Conserved amino-acid residues (six cysteines, one arginine, and one glutamate residue of DEFA5L) show a sequential arrangement that is similar to most other mammalian α-defensins [40].

The transcription of *DEFA5L *could be verified only in the small intestine of the horse [42]. This specific transcription of the peptide and the high similarity to the human α-defensin *DEFA5 *leads to the assumption that it is a Paneth-cell specific peptide. Sequencing of the amplification products revealed two distinct cDNA sequences, *DEFA5L *(already known from the BAC clone) and an unknown sequence denoted as *DEFA1*. The putative propeptides and mature peptides were predicted by comparing the equine sequences to α-defensins of other species. The mature DEFA1 peptide, presumed to be the more active peptide, was recombinantly expressed in *E. coli *and structurally characterized by CD spectroscopy and molecular modeling [42]. It contains a small β-sheet, consisting of three β-strands flanked by large unstructured regions and stabilized by three intramolecular disulfide-bonds as known with other α-defensins.

The antimicrobial activities were comprehensively investigated (Table 3). It was shown that DEFA1 displays a broad spectrum of antimicrobial activity against both fermenting and non-fermenting Gram-negative strains, against Gram-positive cocci, spore-forming bacilli, and the yeast *Candida (C.) albicans*. Remarkably, DEFA1 was shown to be a potent peptide antibiotic against *Rhodococcus (Rh.) equi*, *Strep. equi*, and *Salmonella choleraesuis *[131]. Three different *Rh. equi *strains were analyzed, including the highly infectious *Rh. equi *85FP^+ ^strain. All strains were susceptible at low peptide concentrations.

**Table 3:** Antimicrobial activities of the equine a-defensin DEFA1

Test strain	LD_90 _[μg/mL]	MBC [μg/mL]
Gram-negative bacteria		
		
*E. coli *ATCC 11775	0.8	1.6
*E. coli *ATCC 25922	0.8	1.6
*E. coli *ATCC 35218	0.2	1.6
*E. coli *D31	1.6	3.1
*Kl. pneumoniae *ATCC 13883	12.5	> 25*
*Enterobacter cloacae *ATCC 13047	25	> 25*
*Ps. aeruginosa *ATCC 10145	3.1	> 25*
*Ps. aeruginosa *NCTC 11440	6.2	12.5
*Burkholderia cepacia *ATCC 25416	> 25*	> 25*
*Salmonella choleraesuis *subsp. *typhimurium *serovar Typhimurium	5	10
*P. multocida *subsp. *multocida*	> 10*	> 10*
		
Gram-positive bacteria		
		
*Staph. aureus *ATCC 6538	1.6	3.1
*Staph. aureus *ATCC 12600	1.6	6.2
*Staph. epidermidis *ATCC 14990	3.1	6.2
*Enterococcus faecalis *ATCC 29212	0.8	25
*Enterococcus faecalis *PEG 205 (wild type)	1.6	12.5
*Strept. pyogenes *ATCC 12344	3.1	3.1
*Strept. equi *subsp. *zooepidemicus *	2.5	5
*Strept. equi *subsp. *equi *	2.5	5
*Strept. dysgalactiae *subsp. *equisimilis *	5	10
*Rh. equi *ATCC 33701 P^- ^	2.5	5
*Rh. equi *ATCC 33701 P^+ ^	2.5	5
*Rh. equi *85 FP^+ ^	2.5	5
*Bacillus megaterium *ATCC 14581	0.2	0.8
		
Yeast		
		
*C. albicans *ATCC 24433	3.2	3.2

The antimicrobial activities of DEFA1 were determined by micro dilution assays against different bacterial strains and *C. albicans*. LD_90 _values (90% lethal dose) and MBC values (minimal bactericidal concentration; ≥ 99.9% killing) are given. * = The maximal peptide concentration used in the assay was still below the LD_90 _or MBC, respectively.

Salt sensitivity, as known from other defensins, was also observed with equine DEFA1. Antimicrobial assays performed at higher salt concentrations using *Rh. equi *strains as target organisms resulted in a decreased antimicrobial activity of DEFA1 [131].

DEFA1 rapidly permeates bacterial membranes at acidic and neutral pH [42]. Pore-formation activity was demonstrated. A membrane-selectivity of DEFA1 for negatively charged membranes was examined using liposomes composed of defined phospholipids. An intercalation and insertion of the peptide into the liposome membrane was only observed with phosphatidylglycerol and phosphatidylserine (negative net charge), but not with sphingomyelin, phosphatidylethanolamine, and phosphatidylcholine (neutral net charge) [145]. Therefore, it was concluded that the peptide might act preferentially on prokaryotic but not on eukaryotic membranes.

#### 3.6.3. Repertoire of equine α-defensins

Bruhn et al. analyzed the equine repertoire of α-defensins in more detail [41]. They screened the equine genome in silico for putative α-defensin genes using the known *DEFA5L *and *DEFA1 *sequences as search matrices. Twenty-nine different nucleotide sequences resembling α-defensins were obtained from the "whole genome shotgun"-database of the horse.

**Figure 4:**
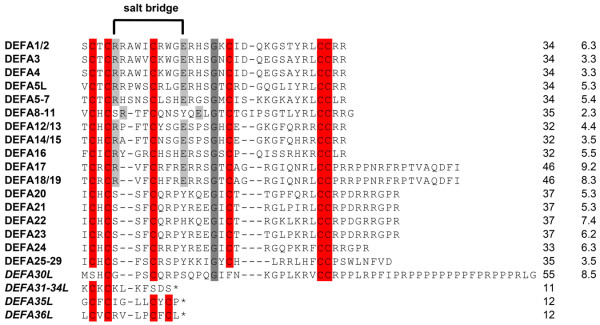
**Comparison of mature a-defensin amino-acid sequences of the horse**. Sequences of a-defensin precursors that share 100% identity within the mature peptide were combined, e.g., DEFA1 and DEFA2 = DEFA1/2. Gaps introduced to maximize amino-acid alignments are indicated by hyphens. Conserved residues are highlighted: cysteine residues are colored red; arginine and glutamic acid, forming an intramolecular salt bridge, are colored light grey; glycine is marked in dark grey. Defensin-like peptides are shown in italics. Premature stop codons are indicated by asterisks. Numbers at the end of each sequence indicate the total number of amino acids followed by the net charge at pH 7.5. DEFA37L is not shown because of a premature stop codon in the propeptide.

Transcriptom analysis with cDNA from the small intestine reveals 38 different equine intestinal α-defensin transcripts. At least 20 of them code for functional peptides due to the fact that typical conserved α-defensin characteristics are present in the primary sequences. These include the conserved cysteine residues, necessary for the typical defensin disulfide-bond connectivity, an arginine and a glutamate residue, forming a conserved salt bridge [146,147], and a highly conserved glycine residue, which is essential for correct folding [148]. At least one of these α-defensin typical characteristics is missing in the mature peptide segment within the remaining 18 sequences. However, this does not inevitably lead to a loss of the biological function of these α-defensins [147]. A sequence alignment of the deduced mature peptides is shown in Figure 4. Several of the equine α-defensins (DEFA17, DEFA18/19, DEFA30L) resemble certain cathelicidins observed in cattle, sheep, and goats [149], because of proline-rich C-terminal extensions. Interestingly, the premature stop codon of two transcripts (*DEFA35L*, *DEFA36L*) is at the same position as that observed for the α-defensin precursors involved in the assembling of cyclic θ-defensins [126] and may indicate an existence of θ-defensins outside primates [41].

A comparison of the genomic sequences and transcripts revealed that ten transcripts lack genomic sequences and 14 genomic sequences lack an appropriate transcript. Moreover, for some transcripts single exons are missing in the genomic database (EquCab 2.0). The authors explain these discrepancies with an incomplete assembly of the horse genome. The transcripts lacking genomic sequences could also be due to copy number variations that have been reported for human α-defensins [150], or an appropriate transcript could be expressed in unexamined tissues and cells.

A comprehensive repertoire of intestinal α-defensins like that in the horse is very rare. The only known organism with a numerically related repertoire of enteric α-defensins is the mouse. At least 23 different peptides are known, named cryptdins and cryptdin-related sequences [151,152]. They were not only found in the small intestine but also in the large intestine, the cecum, and the rectum. The biological relevance of expressing such high amounts of different α-defensin peptides in the gut remains unclear. Probably, the peptides can exhibit different specificities against microorganisms, as observed in mice [151], or the expression level of the α-defensin genes along the intestinal tract shows large variations to optimize the local regulation of bacterial colonization [153]. This may result in an extensive and optimized process of fermentation of the nutriment.

The horse is to date the only known organism expressing α-defensins within the group of *Laurasiatheria*. It remains an open question why for example cattle and dogs presumably lost their complete set of α-defensin genes while in the horse the number increased extensively. Analysis regarding the presence or absence of α-defensins in the closest relatives of the horse like tapir and rhinoceros are missing. Obviously, the horse will play an important role in studies analyzing the evolution or functions of α-defensins.

### 3.7. Hepcidins

Hepcidins are cysteine-rich peptides involved in systemic iron homeostasis by controlling iron absorption and macrophage iron release. In addition they show antimicrobial activities against Gram-positive and Gram-negative bacteria as well as fungi [154,155]. The first hepcidin was identified as an antimicrobial agent in human blood filtrate and denoted LEAP-1 (liver expressed antimicrobial peptide) [154]. As the name implies, hepcidins are predominantly expressed in the liver, but they are also found in much smaller amounts in the heart, brain, lung [154], and body fluids like urine [156]. An upregulation of hepcidins during acute inflammation and in response to experimental inflammatory stimuli as well as IL-6 was observed [155].

Hepcidins are synthesized as precursor peptides; the active form is the mature peptide consisting of 20-30 amino acids. Mature hepcidins share eight conserved cysteine residues across mammalian species, forming four intramolecular disulfide bonds [156]. Basic amino acids confer a positive net charge [155]. The human mature peptide consists of two antiparallel β-sheets linked by a hairpin loop resulting in an amphipathic peptide [157].

In mammals hepcidins share no significant sequence homologies to any of the known antimicrobial peptides, but they resemble structurally some insect defensins and defensin-like drosomycin (from *Drosophila melanogaster*) [155,156]. It was suggested that hepcidin may be a vertebrate counterpart of cysteine-rich antimicrobial peptides produced in the fat body of insects due to the fact that the fat body of insects is the equivalent of the mammalian liver [156].

#### 3.7.1. Equine hepcidin

The equine hepcidin was identified using liver cDNA samples of healthy horses and primer sequences derived from known hepcidin sequences [158]. The equine DNA sequence shows an identity of 74% to *Bos taurus *hepcidin and 70% to hepcidin from *Sus scrofa *and *Canis lupus familiaris*. It was determined that equine hepcidin is located on *Equus caballus *chromosome ten and consists of three exons. Real-time PCR analysis showed a high expression in the liver and a much lower expression (approximately -10^5^) in the cervical spinal cord, cerebral cortex, lung, duodenum, stomach, spleen, kidney, skeletal muscle, and bladder [158].

The equine precursor peptide consists of 86 amino acids, the predicted mature peptide of 25 amino acids. The sequence shows eight conserved cysteine residues, as known from other hepcidins and is 76% identical to bovine, porcine, and human hepcidin [158]. The antimicrobial activities of the equine peptide or its mode of action are hitherto not determined.

## 4. Antimicrobial peptides of vertebrates in practice and clinical studies

In contrast to the great number of studies describing impressive in vitro-activities of antimicrobial peptides, only few reports exist about in vivo-experiments or practical applications. One reason could be that in vivo-studies are much more expensive and sophisticated. Additionally, antimicrobial peptides in general have some remarkable disadvantages that are described here briefly together with some selected successful clinical trials made with antimicrobial peptides of vertebrates. Peptides of the horse have not yet been included in preclinical or clinical studies.

### 4.1. Advantages of antimicrobial peptides as therapeutic drugs in general

*i*, The mode of action finally aims at a fundamental structure of the target cell, the bacterial membrane. The membrane is much more difficult to rearrange than intracellular metabolic pathways. Therefore, the development of resistances is rare.

*ii*, Some antimicrobial peptides simultaneously exhibit antibacterial, antifungal, and antiviral activities. In the case of multiple infections, only one therapeutic agent might be needed. Additionally, antibacterial peptides can act against a broad spectrum of Gram-positive and Gram-negative bacteria in parallel.

*iii*, Many antimicrobial peptides are induced by stimulants. A prophylactic vaccination is possible.

### 4.2. Disadvantages of antimicrobial peptides as therapeutic drugs in general

*i*, Antimicrobial peptides generally are susceptible to proteolytic degradation. Therefore, a systemic application might be difficult to manage.

*ii*, Peptides are cytotoxic against host cells in some cases. Studies regarding cytotoxicity are rare compared to the numerous reports describing antimicrobial activities.

*iii*, Antimicrobial activities are often influenced by salt concentrations, pH-value, and/or plasma proteins. Some peptides with prominent antimicrobial potencies under low-salt conditions lose their activities at physiological salt conditions and/or in serum (due to interactions with serum proteins).

### 4.3. Antimicrobial peptides of vertebrates in human clinical trials

Despite the disadvantages, some antimicrobial peptides have reached clinical trials. Among them is a derivative (MSI-78) of magainin, isolated from the skin of the African clawed frog *Xenopus laevis*, denoted Pexiganan, for the treatment of impetigo contagiosa and diabetic foot ulcer [159-162]. A derivate (IB-367) of pig protegrin, denoted Iseganan, is used for the treatment of oral mucositis [163]. Neuprex (a derivate of the human bactericidal permeability protein, rBPI23) is used for the treatment of sepsis, and Omiganan against catheter associated infections (administered as a 2% gel) [164,165]. Omiganan is a variant of indolicidin from cattle (CPI-226). Actually, only Pexiganan and Omiganan show a significant effectivity compared to conventional antibiotics in the third phase of clinical trials.

Consequently, it seems that the most lucrative application of antimicrobial peptides and proteins is a local treatment rather than a systemic application. Normally, the peptides act in short time intervals and concomitant high concentrations before they become degraded or bound to other peptides.

Recent patents including antimicrobial peptides and possible applications are reviewed in [166].

## 5. Equine candidates for development of therapeutic applications: capabilities and prospects

Equine antimicrobial peptides have not yet been subjected to clinical experiments. Accordingly, data of equine antimicrobial peptides represent basic research. The current knowledge on antimicrobial peptides of the horse is too limited to discuss them as a source for peptide antibiotics in general. Nevertheless, they represent a promising tool to treat infectious diseases of the horses themselves, especially if multiresistant strains are involved. A homologous use of peptide antibiotics minimizes the risk of immunologic incompatibility that might occur in the case of heterologous use. The administration of antimicrobial peptides across species barriers is theoretically possible, but this requires a comprehensive modification of the molecules. Anyway, successful clinical studies in humans performed with several antimicrobial peptides of vertebrates clearly demonstrate that they are suitable for therapeutical applications.

Potentials for practical applications of equine antimicrobial peptides in horses can be deduced from results obtained with antimicrobial peptides in general. Advantageously, the equine innate immune system and its antimicrobial peptides show some remarkable features offering particular chances, for example the high number of α-defensins in the intestine. The probably most promising equine peptides that might influence veterinary medicine are lysozymes, NK-lysin, cathelicidins, and defensins.

### 5.1. Equine lysozyme

Equine lysozyme expression was detected in many different organs of the horse (Figure 1). Its association with horse disease patterns has been proven, albeit by only recording its expression levels. Moreover, the expression and secretion of equine lysozyme by Paneth cells and neutrophils emphasize its implication into the innate immune defense of the horse. However, there is yet no clear suggestion for the therapeutical use of this enzyme in the horse or beyond. Closest to a putative clinical application would be the use of the lysozyme activity as an indicator of inflammation in cases of acute joint injury as suggested by Torbeck and Prieur [59]. Of course this would not address the antimicrobial activity of the EL directly and it is restricted to horses. Another approach that would also not address the classical antimicrobial function is the use of cytotoxic activity. A detailed investigation of the functions and properties of cytotoxic ELOA might be used profitably in various biotechnological applications with the potential to specifically target undesirable cells [70].

Nevertheless, the equine lysozyme is probably one of the most promising candidates for a putative therapeutical use with respect to the knowledge gathered in other species. On the one hand, lysozymes directly mediate resistance to bacterial infections as demonstrated in mice and humans [167-170]. On the other hand, lysozymes possess immunomodulatory, anti-inflammatory functions [171] and they can modulate the microflora of the gut [172,173]. The latter can be easily achieved by oral administration and was found to decrease stress symptoms in hens after vaccination (patent: DE2907236C2). These data clearly represent the potential that equine lysozyme might have.

### 5.2. Equine NK-lysin

As shown by Davis et al. [82], equine NK-lysin is upregulated after intravenous administration of non-viable *Pb. acnes *(see also section 3.2.). *Pb. acnes *has been used as an immunostimulant in horses for almost two decades, and beneficial responses to *Pb. acnes *administration have been characterized by clinical treatment of non-specific respiratory disease [84,174]. The use of *Pb. acnes *combined with an antimicrobial therapy leads to an overall success rate of over 90%, in contrast to a recovery rate of less than 50% if horses are treated with antibiotics alone. The successful clinical trials illustrate that the stimulation of the immune system has a significant influence on the recovery and health of horses. Davis et al. [82] showed that antimicrobial peptides, especially NK-lysin, are included in this process. The development of more specific stimulators influencing the synthesis of antimicrobial peptides could be an attractive possibility for the treatment and prevention of infectious diseases.

The antimicrobial potencies of the equine NK-lysin are hitherto not determined. But with respect to the common high activity against microbes and cancer cells determined in other species one may assume that equine NK-lysin employs an equivalent activity and mode of action.

Of particular interest is that NK-lysin fragments and derivates also show potent antimicrobial activities by concurrent optimization of peptide stability and minimized cytotoxicity. NK-2, a fragment of the porcine NK-lysin, comprising the α-helical, cationic core region, is a very efficient antimicrobial agent and, on the contrary to natural NK-lysin, has only little hemolytic and cytotoxic activities. NK-2 is also able to preferentially kill cancer cells and exhibits antimicrobial activity against intracellular pathogens. It was designated as a promising candidate for clinical applications [175,176] and novel antiinfectives [177].

The importance of equine NK-lysin in the immune regulation of the horse was demonstrated already. The successful investigations of NK-lysins of other species should encourage the equine veterinary researchers to study the equine NK-lysin in more detail.

### 5.3. Equine cathelicidins

The equine cathelicidins are, next to the equine defensins, the most potent antimicrobials with a broad spectrum activity against bacteria. Compared to the defensins they have additional advantages. The cathelicidins eCATH-1 and eCATH-2 are not salt sensitive [35]. The salt and plasma tolerance of these peptides in combination with their high antimicrobial activities make them interesting candidates for further preclinical studies. Especially, the properties of the most bactericidal peptide eCATH-1 are promising. Curiously, eCATH-1 was not present at the peptide level in the horse population analyzed by Scocci et al. [35]. To our knowledge, it is not known whether an active eCATH-1 is present in some breeds. However, the expression of an additional cathelicidin antimicrobial peptide, or the overexpression of a cathelicidin gene protects transgenic mice against bacterial skin infections [178]. It may be interesting to study the inducibility of eCATH-1 in horses which carry functional alleles and whether null alleles are associated with a higher susceptibility for infections. Recent investigations showed that cathelicidins are inducible by vitamin D3. Moreover, the vitamin-D3 pathway was identified as one of the major regulators of cathelicidins [179,180]. This may provide new basic approaches for the therapy of equine infectious diseases.

The possibility for the optimization of equine cathelicidins was impressively demonstrated by Skerlavaj et al. [111]. A slight modification of the eCATH-3 peptide (LLK-eCATH-3) realized by the substitution of only three amino acids caused a higher hydrophobicity and helical content resulting in a dramatic change of the antimicrobial activity. Antibacterial activities of LLK-eCATH-3 were comparable to those of eCATH-1 (eCATH-3 without modifications lacks antibacterial activities) and antifungal effects were noted.

### 5.4. Equine β-defensins

Data about the antimicrobial properties of equine β-defensins are missing. However, one peptide should be mentioned here: the oral β-defensin *DEFB103*. Transcriptional analysis shows that *DEFB103 *is exclusively expressed in the tongue of the horse [145]. It seems to be a specialized peptide that possibly has an optimized spectrum of activity against oral pathogens and prevents the invasion of pathogens into the digestive tract. The human homolog (*DEFB103*, encoding hBD-3) is also synthesized in oral epithelia and was analyzed comprehensively. It shows strong antimicrobial properties within the group of defensins against a broad spectrum of microbes, including bacteria and fungi [181]. It is stable against proteolytic digestion and its activity is almost not affected by physiological salt concentration [182]. Therefore equine DEFB103 might be a promising candidate for initial analysis of equine β-defensins.

### 5.5. Equine α-defensins

The only intensively studied equine α-defensin is DEFA1. The antimicrobial properties are comparable to conventional antibiotics [131] and the spectrum of activity includes numerous bacteria and *C. albicans *[42]. The stability against digestion and cytotoxicity remains unclear, but it was shown that DEFA1 only interacts with negatively charged liposomal membranes serving as model for bacterial membranes. Interestingly, the peptide has a potent antimicrobial activity against typical horse pathogens, most notably *Rh. equi*. DEFA1 kills three different *Rh. equi *strains, including the highly infectious *Rh. equi *85 FP^+^, and the β-lactam antibiotic resistant strains *Rh. equi *ATCC 33701 P^+ ^at comparable peptide concentrations. The efficacy of DEFA1 is higher than that of the antimicrobial peptide magainin II and the conventional antibiotics ampicillin, clarithromycin or rifampicin, typically used for treatment of *Rh. equi *pneumonia [131].

*Rh. equi *mostly infects the lung of the horse, while a potential molecular weapon against the pathogen is located in the intestine. It might be possible to treat the infected lung of foals with DEFA1 applied as an aerosol. Aerosolization methods have not yet been used in horses for administration of antimicrobial peptides, but they were applied for conventional antibiotics [183]. In tuberculosis, a close model for rhodococcosis, nebulization of capreomycin was effective to treat the disease in a guinea pig model [184]. Although nothing is known about the efficacy and required concentration of DEFA1 treatment in the lower airways of horses, nebulization might be a possible application. First investigations are currently being performed using a mouse model with artificial induced lung infections. The focus is on the in vivo-treatment of *Rh. equi *infections, but human pathogens are also being used. If these investigations are successful, further in vivo analysis with horses are conceivable.

Furthermore, DEFA1 is only one candidate out of numerous other potentially functional α-defensins in the intestine of the horse, but nothing is known about their antimicrobial properties [41]. As shown in Figure 4 some of the mature peptides exhibit a high positive charge, which possibly strengthens the interaction between the peptide and the bacterial cell membrane. Some other promising candidates of equine α-defensins are currently synthesized recombinantly to analyze and compare their potencies.

Paneth cell α-defensins like DEFA1 play an important role in infectious diseases of the gut. Studies in humans show an enhanced expression of α-defensin genes in colonic inflammations [185]. Wehkamp et al. discovered a reduced Paneth cell α-defensin synthesis in ileal Crohn disease, a chronic disease of the intestine [186], and Ferguson et al. found that single nucleotide polymorphisms confer susceptibility to inflammatory bowel disease [187].

Inflammatory bowel diseases have also been described in horses, for example the duodenitis/proximal jejunitis syndrome characterized by catarrhal enteritis with mucosal hyperemia or necrosis. Another example is colitis causing intramural edema and hemorrhagic inflammation of the large intestine. Bacteria have been implicated as etiological agents of both diseases. It will be interesting to analyze the relation between enteric infectious diseases of the horse and a possible deficiency or dysregulation of Paneth cell α-defensins. The high quantity of equine intestinal α-defensins and the biological activity of enteric DEFA1 against microorganisms emphasizes the importance of equine intestinal defensins in the protection of the horse against infections of the intestinal tract and the regulation of the intestinal microbiome, respectively.

Whether copy number variations, nucleotide polymorphisms, or functional mutations of equine α-defensins influence health or performance of horses is unknown. Further investigations are not only of interest for veterinary science and practice, but rather also for animal breeders who are interested in a marker-based selection to preserve and enhance equine health.

## Conclusions

With the objective of discovering new antimicrobial agents in fighting the threatening rise of multidrug-resistant pathogens, so-called super-bugs, antimicrobial peptides and proteins appear most promising. Despite millions of years of evolution, microorganisms failed to develop common resistance strategies protecting them against these molecules. The number of investigations on the identification and isolation of equine antimicrobial peptides during the last decades is pretty small. But recent studies clearly show that the horse represents a rich source of antimicrobial peptides and proteins, especially defensins, of potential therapeutic use.

## Competing interests

The authors declare that they have no competing interests.

## Authors' contributions

OB developed the structural design of the review, coordinated the work and did together with SJ the major part of drafting of the manuscript. JG and IC participated in drafting of the manuscript and revised the manuscript critically. All authors read and approved the final manuscript.
